# Effect of herbal compounds on coronavirus; a systematic review and meta-analysis

**DOI:** 10.1186/s12985-022-01808-z

**Published:** 2022-05-21

**Authors:** Mina Mobini Kesheh, Sara Shavandi, Niloofar Haeri Moghaddam, Moazzameh Ramezani, Fatemeh Ramezani

**Affiliations:** 1 Department of Virology, School of Medicine, Iran University of Medical Sciences, Tehran, Iran; 2 Department of Medical Nanotechnology, Faculty of Advanced Technologies in Medicine, Iran University of Medical Sciences, Tehran, Iran; 3 Texas A&M AgriLife Research and Extension Center, Uvalde, TX USA; 4 Physiology Research Center, Iran University of Medical Sciences, Tehran, Iran; 5 Department of Industrial and Environmental Biotechnology, National Institute of Genetic Engineering and Biotechnology, Tehran, Iran

**Keywords:** Coronaviridae, Herbal medicine, Systematic review, Treatment, Alkaloid

## Abstract

**Background:**

The new coronavirus (COVID-19) has been transmitted exponentially. Numerous studies have been performed in recent years that have shown the inhibitory effect of plant extracts or plant-derived compounds on the coronavirus family. In this study, we want to use systematic review and meta-analysis to answer the question, which herbal compound has been more effective?

**Main body:**

The present study is based on the guidelines for conducting meta-analyzes. An extensive search was conducted in the electronic database, and based on the inclusion and exclusion criteria, articles were selected and data screening was done. Quality control of articles was performed. Data analysis was carried out in STATA software.

**Conclusion:**

Due to the variety of study methods, definitive conclusions are not possible. However, in this study, we attempted to gather all the available evidence on the effect of plant compounds on SARS-COV-2 to be used for the development and use of promising antiviral agents against this virus and other coronaviruses. Trypthantrin, Sambucus extract, *S. cusia* extract, Boceprevir and Indigole B, dioica agglutinin urtica had a good effect on reducing the virus titer. Also among the compounds that had the greatest effect on virus inhibition, Saikosaponins B2, SaikosaponinsD, SaikosaponinsA and Phillyrin, had an acceptable selectivity index greater than 10. Andrographolide showed the highest selectivity index on SARS-COV-2. Our study confirmed insufficient data to support alkaloid compounds against SARS-COV-2, and the small number of studies that used alkaloid compounds was a limitation. It is recommended to investigate the effect of more alkaloid compounds against Corona virus.

## Introduction

The outbreak of the new coronavirus (COVID-19) originated in Wuhan, China in December 2019 and has affected many countries around the world. As of March 26, the World Health Organization (WHO) has announced in detail that the disease has spread to 197 countries. Most people infected with the COVID-19 virus experience mild to moderate respiratory illness and recover without special treatment [15, 58]. The elderly and those with underlying medical problems such as cardiovascular disease, diabetes, chronic respiratory disease, and cancer develop serious illness [5, 17].

For providing the best immunization to the community against this virus, alongside developed vaccines, different drugs are still needed for coronavirus inhibition [49]. Remdesivir (Veklury) is currently the only FDA approved drug to treat coronavirus disease. This confirmation was based on findings that hospitalized patients who received Remdesivir recovered faster. Many clinical trials are currently underway to evaluate other potential therapies, such as monoclonal antibodies to COVID-19. Researchers are also testing older drugs (commonly used to treat other diseases) to see if they work for COVID-19.

Plants have beneficial biomedical effects due to their natural properties [33, 42]. Plants are inexpensive and available sources of medicinal compounds that by changing the growth conditions and the effect of various stimulants, the production of medicinal molecules and their effect can be increased several times [3, 12, 41, 43]. The antiviral effects of many plants have been proven. Of course, plants that have previously had an inhibitory effect on the coronavirus family or inhibited the ACE2 enzyme may help inhibit new coronavirus or symptomatic therapy [39].

Traditional herbal medicines have been used since the early days of COVID-19 in China. These traditional drugs have been shown to improve 90% of the 214 patients [14]. Some traditional herbal therapies stopped SARS-COV-2 infection in healthy people and improved the health status of patients with mild or severe symptoms [14, 54]. Traditional Chinese medicine known as Shu Feng Jie Du and Lianhuaqingwen, which have been effective against previous influenza A (H1N1) or SARS-CoV-1 [30], have been recommended. The use of traditional medicines in COVID-19 treatment and prevention guidelines was prepared by a team from Wuhan University's Zhongnan Hospital. Several methods using herbs have been suggested to prevent COVID-19. In addition, for the treatment of the disease, experts recommended the use of different herbal mixtures according to the stage of the disease [19]. Evidence suggests that herbal remedies may be effective in decreasing and managing of COVID-19 risk [13]. Despite many primary study researches, there is no a systematic review article that compare the effects of all studied compounds on the SARS-COV-2 by more details and it can be useful for researchers in this field.

In this study, we conducted a systematic review and meta-analysis on herbal compounds against coronavirus family, which may have the potential in treating COVID19 infection. The purpose of this study is to better understand current compounds in research into the development of new antiviral agents against SARS-COV-2 from plant sources. The findings of this study can help to provide up-to-date knowledge about the antiviral potential against SARS-COV-2 in medicinal plants and to utilize existing knowledge gaps to improve future research by identifying areas for greater focus.

## Method

The present study is designed based on the PRISMA guidelines for systematic review. The present study investigated the inhibitory effect of plant compounds on the coronaviruses family.

### Search strategy

An extensive search of the Medline electronic database, ISI Web of Science, EMBASE, and Scopus was conducted through April 2021. The search strategy was based on the Table 1. Keywords have been selected as widely as possible so that a study is not omitted. To find additional articles or unpublished data, hand-search was performed in the list of relevant articles and related journals.

**Table 1:** Keywords for search of the databases

(("Coronavirus"[MeSH Terms] or "COVID-19" [MeSH Terms] or "Deltacoronavirus"[MeSH Terms] or "Deltacoronavirus"[MeSH Terms] or "Munia coronavirus HKU13"[TIAB] or "Middle East Respiratory Syndrome Coronavirus"[MeSH Terms] or "MERS-COV-2"[TIAB] or "MERS Virus"[TIAB] or "MERS Viruses"[tiab] or "Virus, MERS"[tiab] or "Viruses, MERS"[tiab] or "Coronavirus NL63, Human"[MeSH Terms] or "HCoV-NL63"[tiab] or "Human Coronavirus NL63"[tiab] or "Coronavirus NL63, Human"[tiab] or "Coronavirus Infections"[MeSH Terms] or "Coronavirus Infection*"[tiab] or "Coronavirus*"[tiab] or "SARS Virus"[MeSH Terms] or "Severe Acute Respiratory Syndrome Virus"[tiab] or "SARS-Related Coronavirus"[tiab] or "SARS-CoV"[tiab] or "SARS Coronavirus"[tiab] or "Coronavirus, SARS-Associated"[tiab] or "Alphacoronavirus"[MeSH Terms] or "Alphacoronavirus*"[tiab] or "Rhinolophus bat coronavirus HKU2"[tiab] or "Miniopterus bat coronavirus HKU8"[tiab] or "2019 novel coronavirus infection"[tiab] or "coronavirus disease 2019"[tiab] or "coronavirus disease-19"[tiab] or "2019-nCoV disease"[tiab] or "2019 novel coronavirus disease"[tiab] or "2019-nCoV infection"[tiab] or "Coronavirus 229E, Human"[tiab] or "HCoV-229E"[tiab] or "Human Coronavirus 229E"[tiab] or "Betacoronavirus"[tiab] or "Betacoronaviruses"[tiab] or "Pipistrellus bat coronavirus HKU5"[tiab] or "Human coronavirus HKU1"[tiab] or "HCoV-HKU1 "[tiab] or "Rousettus bat coronavirus HKU9"[tiab] or "Betacoronavirus1"[tiab] or "Human enteric coronavirus"[tiab] or "Human enteric coronaviruses"[tiab] or "Coronaviruses"[tiab] or "Deltacoronavirus*"[tiab] or "Coronavirus Infections"[tiab] or "Coronavirus Infection"[tiab] or "Infection, Coronavirus"[tiab] or "Infections, Coronavirus"[tiab] or "Middle East Respiratory Syndrome"[tiab] or "SARS Virus"[tiab] or "SARS Related Coronavirus"[tiab] or "Coronavirus, SARS"[tiab] or "Severe acute respiratory syndrome-related coronavirus"[tiab] or "Coronavirus, SARS-Associated"[tiab] or "SARS Associated Coronavirus"[tiab] or "Alphacoronavirus*"[tiab] or "COVID19"[tiab] or "2019 novel coronavirus infection"[tiab] or "2019 novel coronavirus disease"[tiab] or "Betacoronaviruses"[tiab] or "Betacoronavirus 1 "[tiab] or "Human enteric coronavirus*"[tiab]) AND ("Plants"[MeSH Terms] or "Plant Mucilage"[MeSH Terms] or "Plant Gums"[MeSH Terms] or "Plant Exudates"[MeSH Terms] or "Plant Lectins"[MeSH Terms] or "Plant Oils"[MeSH Terms] or "Plant Proteins"[MeSH Terms] or "Resins, Plant"[MeSH Terms] or "Plant Extracts"[MeSH Terms] or "Flowers"[MeSH Terms] or "Plants, Medicinal"[MeSH Terms] or "Plant*" [TIAB] or "Plant Mucilage"[tiab] or "Plant Gums"[tiab] or "Plant Exudates"[tiab] or "Plant Lectins"[tiab] or "Plant Oils"[tiab] or "Plant Proteins"[tiab] or "Resins, Plant"[tiab] or "Plant Extracts"[tiab] or "Flowers"[tiab] or "Plants, Medicinal"[tiab] or "Medicinal Plant"[tiab] or "Plant, Medicinal"[tiab] or "Pharmaceutical Plant*"[tiab] or "Plant, Pharmaceutical"[tiab] or "Plants, Pharmaceutical"[tiab] or "Healing Plants"[tiab] or "Healing Plant"[tiab] or "Plant, Healing"[tiab] or "Medicinal Herbs"[tiab] or "Herb, Medicinal"[tiab] or "Medicinal Herb"[tiab] or "Herbs, Medicinal"[tiab] or "herbal medicine"[tiab] or "Leave, Plant"[tiab] or "Plant Leave*"[tiab] or "Plant Leaf"[tiab] or "Leaf, Plant"[tiab]))	

### Inclusion and exclusion criteria

Controlled in-vitro and in-vivo studies were selected to investigate the inhibitory effect of plant compounds against each of the coronaviruses. Controlled studies are studies that, in addition to a group treated with a plant composition, also have a control group without treatment. No time or language restrictions were imposed. Because most viral studies are performed in an in-vitro model, the target population for this study is SARS-COV-2 virus-infected cells.In the present study, short articles and letters to the editor were not examined. Review articles were not included in the study.

### Outcomes

In the present study, the Selectivity Index (SI) (the CC50/EC50 ratio) was extracted from articles. CC50 is the concentration of compound required to reduce host cell viability by 50% and EC50 is the concentration of compound required to reduce virus function by 50%. In addition, studies that have examined each of the factors of inhibition of virus and virus titer are included in the meta-analysis.

The extracted articles were evaluated independently by two researchers and the data were recorded in the data extraction form. In case of disagreement between two researchers, the third person studied the findings and resolved the existing disagreement by discussing and exchanging views with the other two researchers. Data collection was done without prejudice and restrictions on the author, journal, organization or organ. The results of a systematic search in this study were recorded in a checklist designed based on PRISMA statement guidelines. The extracted data included general information of the article (author name, year of publication), information related to the design of the study, characteristics of the studied host such as cell type, as well as characteristics of the studied plant such as plant name and strain. When the consequences and values ​​to be evaluated are reported in several stages, the last evaluation time was entered into the research. If the results were presented in the form of graphs, the data extraction method was used.

### Quality control

The evaluation of the quality of the studies included in this study has been done according to the methods described in published articles [18, 28]. Eight groups of criteria include 20 items were examined (exclusions, randomization, blinding, sample size, figures and statistical representation of data, definition of statistical methods and measures, implementation of statistical methods and measures, reagents and cells). These criteria were extracted from the articles by the twenty separate cases mentioned below:

(1) Samples that were excluded from the analysis.

(2) Which method of randomization was used to determine how samples were allocated to experimental groups?

(3) Whether the investigator was blinded to the group allocation during the experiment and/or when assessing the outcome,

(4) How the sample size was chosen to ensure adequate power to detect a pre-specified effect size.

(5) Exact sample size (n) for each experimental group/condition was given as a number, not a range.

(6) Whether the samples represented technical or biological replicates.

(7) A statement of how many times the experiment was replicated.

(8) Results were defined as a median or average.

(9) Error bars were defined as SD., SEM. or CI.

(10) Common statistical tests (such as t-test, simple χ2 tests, Wilcoxon and Mann–Whitney tests, or any form of ANOVA testing). If not a common test, is the test is described in the methods section.

(11) If the statistical test used was a t or z test, was it reported as one sided or two sided.

(12) Adjustments for multiple comparisons were applied where appropriate.

(13) The statistical test results (e.g., P values, F statistic etc.) were presented.

(14) The authors show that their data met the assumptions of the tests.

(15) An estimate of variation is reported for each group of data.

(16) The variance between the groups that were statistically compared was comparable (difference less than two-fold).

(17) Every antibody used in the manuscript been characterized by either citation, catalog number, clone number or validation profile,

(18) The source of all cell lines was provided.

(19) The authors reported whether the cell lines used have been recently authenticated.

(20) The authors reported whether the cell lines have recently been tested for contamination (within 6 months of use).

### Meta-analysis

All analyzes were performed using Stata 14. Data were obtained from the mean of different ratios between experimental and control groups. The random effect model was used. Subgroup analysis was performed for the chemical structure of the plant composition used, viral subtype and cell line type studied. P values were reported by testing the statistical hypothesis at the level of 0.05 bilaterally.

## Results

### Applying exclusion criteria

**Fig. 1:**
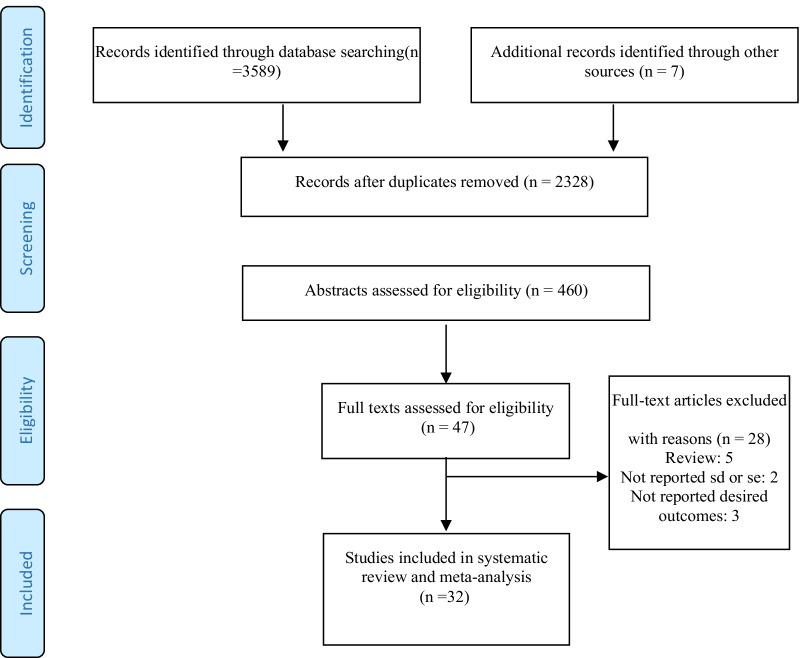
PRISMA flow chart for a systematic review with database search details, number of abstracts and retrieved full text displayed

To reach the studies that met our inclusion criteria (see Fig. 1), we searched the articles and identified 3,589 studies that appeared to be relevant. 1268 studies were duplicates and were omitted. Of the remaining 2328 studies, 47 articles remained after reviewing titles and abstracts. After reviewing the texts of the articles, 15 articles were deleted and 32 articles remained in the study.

### Characteristics of included studies

Table 2 shows the characteristics of the articles included in this study. 15 articles were on SARS-COV, 9 articles were on SARS-COV-2, 6 articles were on HCOV, 3 articles were on IBV, 2 articles were on PEDV and 2 articles were on MERS-COV-2. SI were extracted from 23 studies and EC50 obtained from 16 articles. In 10 articles virus inhibition and in 8 articles virus titer measurements were reported. Other characteristics of the articles such as host cell type, strain and plant genus, drug composition are listed in Table 2.

**Table 2:** Information about the articles included in this study NR: Not reported, SI: selectivity index, MRC5: human embryonic lung fibroblast

Ref	Virus strain/host	Drug name/plant	Main outcomes	exposure time/hours	SI(CC50/IC50)	EC50	
[37]	MERS-COV-2/MRC-5	Silvestrol/ Meliaceae	SI, Virus titer	24 h	> 7690	0.0013 µM/L	
	HCoV-229E/ MRC-5				> 3330	0.003 µM/L	
	HCoV-229E/ PBMCs				> 350	0.0028 µM/L	
	HCoV-229E/ Huh-7				> 0.75	0.040 µM/L	
[9]	HCoV-229E/ MRC-5	Saikosaponins B2/ Bupleurum	SI, EC50, Virus inhibition	96 h	221.9	1.7 ± 0.1 µM/L	
		Saikosaponin A/ Bupleurum			26.6	8.6 ± 0.3 µM/L	
		Saikosaponin C/ Bupleurum			19.2	19.9 ± 0.1 µM/L	
		Saikosaponin D/ Bupleurum			13.3	13.2 ± 0.3 µM/L	
[7]	SARS-COV strain FFM 1/ African green monkey kidney cell lines Vero	Extract/Yin-Chiau-San	SI, EC50	72 h	> 1	> 500(µg/ml)	
		Extract/ Pu-Zhi-Siau-Du-Yien			> 2	240(µg/ml)	
		Extract/ Ger-Gern-Hwang-Lein			> 3	134(µg/ml)	
		Extract/ Sang-Zhiu-Yien			> 1	349(µg/ml)	
		Extract/ Huang-Lein-Zhei-Du-Tang			> 1	369(µg/ml)	
		Extract/ *Toona sinensis* leaves			17	30(µg/ml)	
		Extract/ *Toona sinensis* leaves			> 13	37 (µg/ml)	
		Extract /Amaryllidaceae			370	2.4 (± 0.2) (µg/ml)	
[25]	SARS-COV (BJ-001)/ Vero E6 cells	Artemisia annua	SI, EC50, Virus inhibition,	72 h	31	34.5(± 2.6) (µg/ml)	
		Pyrrosia lingua			55	43.2(± 14.1) (µg/ml)	
		Lindera aggregate			16	88.2(± 7.7) (µg/ml)	
	SARS-COV (BJ-002)/ Vero E6 cells	Extract/ Lycoris radiata /Amaryllidaceae			422	2.1 (± 0.2) (µg/ml)	
		Artemisia annua			27	39.2 (± 4.1) (µg/ml)	
		Pyrrosia lingua			59	40.5 (± 3.7) (µg/ml)	
		Lindera aggregate			17	80.6 (± 5.2) (µg/ml)	
[53]	HCoV-NL63/ LLC-MK2 cells, Calu-3 cells	Caffeic acid /Adoxaceae Chlorogenic acid/Adoxaceae Gallic acid/Adoxaceae	Virus inhibition, Virus titer		NR	NR	
[20]	SARS-COV / Vero E6 cells	Lectin (Man-specific agglutinins)(APA/ Alliaceae	SI, EC50	72	> 222.2	0.45 ± 0.08(µg/ml)	
		Mannose-specific agglutinins( HHA)			> 31.3	3.2 ± 2.8(µg/ml)	
		Mannose-specific agglutinins( GNA)			> 16.1	6.2 ± 0.6(µg/ml)	
		Mannose-specific agglutinins( NPA)			> 17.5	5.7 ± 4.4(µg/ml)	
		Mannose-specific agglutinins( LRA)			> 2.1	48(µg/ml)	
		Mannose-specific agglutinins(AUA)			> 5.5	18 ± 4(µg/ml)	
		Mannose-specific agglutinins( CA)			> 20	4.9 ± 0.8(µg/ml)	
		Mannose-specific agglutinins( LOA)			> 45.5	2.2 ± 1.3(µg/ml)	
		Mannose-specific agglutinins( EHA)			> 55.5	1.8 ± 0.3(µg/ml)	
		Mannose-specific agglutinins( TLMI)			> 2.3	22 ± 6(µg/ml)	
		Mannose-specific agglutinins( Morniga M II)			> 62.5	1.6 ± 0.5(µg/ml)	
		GlcNAc-specific agglutinins Nictaba			> 58.8	1.7 ± 0.3(µg/ml)	
		(GlcNAc)n-specific agglutinins UDA			> 76.9	1.3 ± 0.1(µg/ml)	
		Gal-specific agglutinins Morniga G II			> 2	50 ± 13(µg/ml)	
		Man/Glc-specific agglutinins Cladistris			> 13.5	7.4 ± 0.2(µg/ml)	
		Gal/GalNAc specific agglutinins -PMRIP m			> 5.5	18 ± 13(µg/ml)	
		GalNAc (> Gal) specific agglutinins/ ML III			> 12.6	28 ± 11(µg/ml)	
		GalNAcα(1,3)Gal > GalNAc > Gal-specific agglutinins/IRA			22.7	2.2 ± 0.9(µg/ml)	
		GalNAcα(1,3)Gal > GalNAc > Gal-specific agglutinins/IRA			8.2	4.4 ± 3.1(µg/ml)	
		GalNAcα(1,3)Gal > GalNAc > Gal-specific agglutinins/IRA			16.2	3.4 ± 2.0(µg/ml)	
		Man/GalNAc-specific agglutinins/ TL C II			> 1.3	38 ± 0(µg/ml)	
[22]	SARS-COV, Toronto-2 v2147/ Vero 76	Lectin (N-acetylglucosamine)/ Urticaceae	SI, Virus titer	72 h	54.2 ± 52.5	NR	
	SARS-COV, Urbani/ Vero 76				10.2 ± 5.6	NR	
	SARS-COV, Mouse-adapted virus/ Vero 76				42.8 ± 47.5	NR	
	SARS-COV, Frankfurt v1940/ Vero 76				5.5 ± 2.0	NR	
	SARS-COV, Hong Kong v2157/ Vero 76				8.6 ± 1.1	NR	
[23]	Vero-adapted Beaudette IBV/ Vero	Ethanol extract/ Lamiaceae	SI, EC50, Virus titer	72 h	67.5	0.004 (µg/ml)	
	Vero-adapted Beaudette IBV/ Satureja montana				17	0.044(µg/ml)	
	Vero-adapted Beaudette IBV/Origanum vulgare				65	0.008(µg/ml)	
	Vero-adapted Beaudette IBV/Mentha piperita				67.5	0.015(µg/ml)	
	Vero-adapted Beaudette IBV/Melissa officinalis				39.3	0.010(µg/ml)	
	Vero-adapted Beaudette IBV/Hyssopus officinalis,				8.4	0.076(µg/ml)	
	Vero-adapted Beaudette IBV/ Salvia officinalis,				36.7	0.003(µg/ml)	
					17.1	0.017(µg/ml)	
	Vero-adapted Beaudette IBV/Desmodium canadense						
[26]	SARS-COV pseudovirus/ HEK293T-ACE2	Sanguisorba/ Rosaceae	Virus inhibition,		NR	NR	
[35]	HCo-229E/ Epithelial colorectal adenocarcinoma cells(Caco-2)	Extract/ Pelargonium sidoides/ Geraniaceae	SI, EC50, Virus titer	72 h	> 2.3	NR	
[51]	SARS-COV / Vero E6	Ferruginol	SI, EC50,	72 h	58	1.39 (µM)	
		Dehydroabieta-7-one			76.3	4	
		Sugiol			NR	n.t	
		Cryptojaponol			< 7.9	> 10	
		8â-hydroxyabieta-9(11),13-dien-12-one			> 510	1.47	
		7â-hydroxydeoxycryptojaponol			111	1.15	
		6,7-dehydroroyleanone			16.2	5.55	
		3â,12-diacetoxyabieta-6,8,11,13-tetraene			193	1.57	
		Pinusolidic acid			159	4.71	
		Forskolin			89.8	7.5	
		Cedrane-3â,12-diol			NR	> 0	
		α -cadinol			17.3	4.44	
		Betulinicacid			< 15	> 10	
		Betulonic acid			180	0.63	
		Hinokinin			NR	> 10	
		Savinin			> 667	1.13	
		4,4 ′ -O-benzoylisolaricires-inol			N.C	n.t	
		Honokiol			13.7	6.50	
		Magnolol			18	3.80	
		Curcumin			NR	> 10	
[52]	SARS-COV / Vero E6	Supernatant of Cibotium barometz	SI, EC50	72 h	> 59.4	8.42 (μg/ml)	
		70% ethanol precipitated fraction of Cibotium barometz			NR	> 10(μg/ml)	
		Dried rhizome of Gentiana scabra			> 57.5	8.70(μg/ml)	
		The tuber of Dioscorea batatas			> 62.0	8.06(μg/ml)	
		The dried seed of Cassia tora			> 59.3	8.43(μg/ml)	
		The dried stem, with leaf of Taxillus chinensis			> 92.8	5.39(μg/ml)	
[55]	PEDV/ Vero cells	Oleanane triterpenes2 / Theaceae	SI, EC50	72 h	13.39 ± 0.67	1.94 ± 0.39 (µM/L)	
		Oleanane triterpenes3 / Theaceae			5.75 ± 0.75	1.09 ± 0.22 (µM/L)	
		Oleanane triterpenes6 / Theaceae			44.54 ± 8.34	0.28 ± 0.09 (µM/L)	
		Oleanane triterpenes7 / Theaceae			7.99 ± 0.28	0.91 ± 0.07 (µM/L)	
		Oleanane triterpenes8 / Theaceae			12.98 ± 2.34	0.06 ± 0.02 (µM/L)	
		Oleanane triterpenes9 / Theaceae			32.72 ± 6.22	0.28 ± 0.11 (µM/L)	
		Oleanane triterpenes10 / Theaceae			9.4 ± 1.04	2.90 ± 0.25 (µM/L)	
		Oleanane triterpenes11 / Theaceae			14.75 ± 1.62	0.93 ± 0.22 (µM/L)	
		Oleanane triterpenes13 / Theaceae			6.68 ± 0.14	0.34 ± 0.01 (µM/L)	
		Oleanane triterpenes15 / Theaceae			6.42 ± 0.58	3.70 ± 0.68 (µM/L)	
[56]	Porcine epidemic diarrhea virus (PEDV)/ Vero cells	Coumarins/ Saposhnikovia divaricate1/ Umbelliferae	SI, EC50	72 h	> 6.25 ± 0.85	16.25 ± 1.97 (µM/L)	
		Coumarins/ Saposhnikovia divaricate2/ Umbelliferae			> 5.85 ± 0.80	17.36 ± 2.12 (µM/L)	
		Coumarins/ Saposhnikovia divaricate3/ Umbelliferae			> 5.11 ± 0.47	19.70 ± 1.66 (µM/L)	
		Coumarins/ Saposhnikovia divaricate4/ Umbelliferae			4.07 ± 0.25	3.84 ± 0.45 (µM/L)	
		Coumarins/ Saposhnikovia divaricate5/ Umbelliferae			> 23.90 ± 4.11	4.28 ± 0.64 (µM/L)	
		Coumarins/ Saposhnikovia divaricate6/ Umbelliferae			7.67 ± 0.04	1.09 ± 0.06 (µM/L)	
		Coumarins/ Saposhnikovia divaricate7/ Umbelliferae			8.21 ± 0.40	1.22 ± 0.09 (µM/L)	
		Coumarins/ Saposhnikovia divaricate8/ Umbelliferae			7.89 ± 0.97	0.60 ± 0.03 (µM/L)	
		Coumarins/ Saposhnikovia divaricate9/ Umbelliferae			> 5.58 ± 0.41	18.00 ± 1.25 (µM/L)	
[57]	IBV/ Vero cells	Houttuynia cordata (Saururaceae)	Virus inhibition	1 h	NR	NR	
		Glycyrrhizinate diammonium (GD)					
	IBV/ chicken embryo kidney (CEK) cells	Houttuynia cordata (Saururaceae)					
		Glycyrrhizinate diammonium (GD)					
[8]	(IBV) a chicken coronavirus/ Vero cells	Rhodiola rosea, Nigella sativa, Sambucus nigra	Virus titer	3d	NR	NR	
[36]	MERS-COV-2 strain EMC/2012/ MRC-5	Griffithsin (GRFT)/ Wrangeliaceae	Virus titer	45 h	NR	NR	
[59]	SARS-COV strain PUMC01 F5 / VeroE6	Forsythiae Fructus	SI	72 h	1.4	NR	
		Scutellariae Radix			1.0	NR	
		Astragali Radix			1.7	NR	
		Bupleuri Radix			< 1	NR	
		Glycyrrhizae Radix			< 1	NR	
		Cinnamomi Cortex (CCE)			6.6	NR	
		Ethanol extract of CC (Fr.1)			5.2	NR	
		Butanol fraction of CC (Fr.2)			5.5	NR	
		Aqueous fraction of CC (Fr.3)			3.9	NR	
		Ethylacetate fraction of CC (Fr.4)			3.4	NR	
		Caryophylli Flos (CFE)			12.9	NR	
		Ethanol extract of CF (Fr.1)			5.4	NR	
		Butanol fraction of CF (Fr.2)			20.9	NR	
		Aqueous fraction of CF (Fr.3)			23.4	NR	
		Ethylacetate fraction of CF (Fr.4)			7.3	NR	
[50]	SARS-COV-2/ Vero E6	Artemisinin(LG0019527)	Virus inhibition	1 h	NR	NR	
[48]	HCoV-NL63/ LCC-MK2	Trypthantrin	Virus titer	48 h	NR	NR	
		Indigodole B					
[6]	SARS-COV‑2/ Vero E6 cells	Arteether	SI, EC50,	24 h	6.42	31.86 ± 4.72 µM	
		Artemether				3.13	73.8 ± 26.91
		Artemisicacid				3.3	> 100
		Artemisinin				3.11	64.45 ± 2.58
		Artemisone				4.03	49.64 ± 1.85
		Dihydroartemisinin				2.38	13.31 ± 1.24
		Artesunate				5.1	12.98 ± 5.3
		Arteannuin				7	10.28 ± 1.12
		lumefantrine			4.4	23.17 ± 3.22	
[46]	SARS-COV-2/ HepG2	Andrographolide	Virus inhibition	48 h	2398	NR	
	SARS-COV-2/ imHC				1310	NR	
	SARS-COV-2/ HK-2				1003	NR	
	SARS-COV-2/ Caco-2				1538	NR	
	SARS-COV-2/ Caco-3				1707	NR	
	SARS-COV-2/ SH-SY5Y				388	NR	
[40]	SARS-COV‑2/ Vero E6	Resveratrol	EC50	2 h	NR	66 µM	
		Pterostilbene				19 µM	
[10]	SARS-COV-2/ Vero E6	Homorringtonine	EC50	48 h	NR	2.55 μM	
		Emetine				0.46 μM	
[31]	SARS-COV-2/ Vero E6	Phillyrin (KD-1)	Virus inhibition	72 h	30.66	NR	
	HCoV-229E/ Vero E6				16.02		
[32]	SARS-COV-2/ Vero E6	Liu Shen capsule	Virus inhibition	72 h	8.18	NR	
[38]	SARS-COV/ Vero E6	Griffithsin	SI, EC50	3d	> 164	0.61 µg/ml	
[24]	SARS-COV-2/ Vero E6	Corilagin (RAI-S-37)	SI, EC50	24 h	NR	0.13 μmol/L	
[45]	SARS-COV-2/ Huh-7	EGYVIR	Virus inhibition	3d	NR	NR	
[11]	SARS-COV/ Vero cells	Glycyrrhizin	SI, EC50	72 h	> 67	300 µg/ml	
[21]	HCoV-C43/ MRC-5 human lung cell	Tetrandrine	SI	4d	40.19	NR	
		Fangchinoline			11.46	NR	
		Ceparanthine			13.63	NR	
[29]	SARS-COV/ Vero	L. nobilis	SI	48 h	4.2	NR	
		T. orientalis			3.8	NR	
		J. oxycredrus ssp. Oxycedrus			3.7	NR	
		Pyramidalis			1.5	NR	
		P. palaestina			> 1	NR	
		S. officinalis			> 1	NR	
		S. thymbra			NR	NR	
		Acyclovir			NR	NR	
		Glycyrrhizin				1.2	NR

In herbal medicine research, it is common to observe multiple medicinal properties of a plant. It is now well understood that a plant may contain a wide range of chemicals, and have different effects on the virus and the host cell [27]. In this study, SI was one of the indicators extracted from the articles. Awouafack et al. Recommended a SI ≤ 10 acceptance criterion for selecting an active sample [4]. In this study in addition to inhibiting the virus, and reducing the virus titer, the amount of SI was extracted from articles (Table 2).

As shown in Table 2, among all plant compounds, Silvesterol has an SI > 7690 on MERS-COV-2 virus in the host of infected human embryonic lung fibroblast (MRC-5) cell, which has the highest SI. In rank 2, the SI of Saikosaponins B2 was 221 on the HCOV strain.

**Fig. 2:**
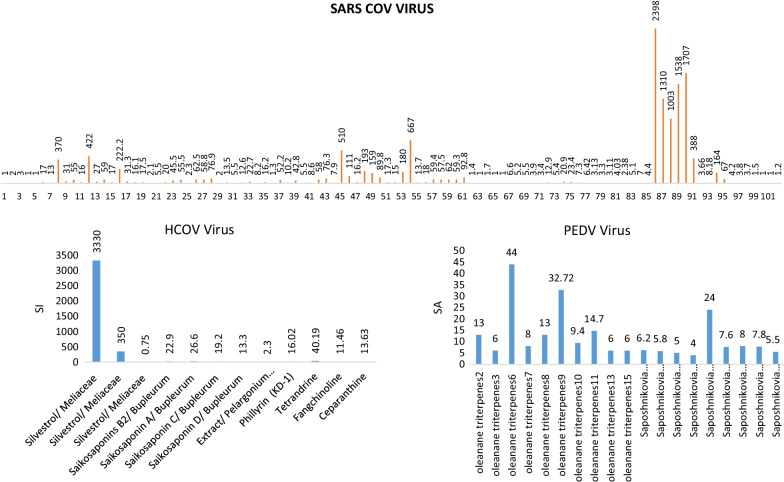
SI value for different compounds on different strains of Coronavirus family. 1. Extract/Yin-Chiau-San, 2. Extract/ Pu-Zhi-Siau-Du-Yien, 3. Extract/ Ger-Gern-Hwang-Lein, 4. Extract/ Sang-Zhiu-Yien, 5. Extract/ Huang-Lein-Zhei-Du-Tang, 6. Extract/ Toona sinensis leaves, 7. Extract/ Toona sinensis leaves, 8. Extract /Amaryllidaceae, 9. Artemisia annua, 10. Pyrrosia lingua, 11. Lindera aggregate, 12. Lycoris radiata, 13. Artemisia annua, 14. Pyrrosia lingua, 15. Lindera aggregate, 16. Lectin (Man-specific agglutinins)(APA), 17. Mannose-specific agglutinins( HHA), 18. Mannose-specific agglutinins( GNA), 19. Mannose-specific agglutinins( NPA), 20. Mannose-specific agglutinins( LRA), 21. Mannose-specific agglutinins(AUA), 22. Mannose-specific agglutinins( CA), 23. Mannose-specific agglutinins( LOA), 24. Mannose-specific agglutinins( EHA), 25. Mannose-specific agglutinins (TLMI), 26. Mannose-specific agglutinins( Morniga M II), 27. GlcNAc-specific agglutinins Nictaba, 28. (GlcNAc)n-specific agglutinins UDA, 29. Gal-specific agglutinins Morniga G II, 30. Man/Glc-specific agglutinins Cladistris, 31. Gal/GalNAc specific agglutinins –PMRIP, 32. GalNAc (> Gal) specific agglutinins/ ML III, 33. GalNAc α (1,3)Gal > GalNAc > Gal-specific agglutinins/IRA, 34. GalNAc α (1,3)Gal > GalNAc > Gal-specific agglutinins/IRA, 35. GalNAc α (1,3)Gal > GalNAc > Gal-specific agglutinins/IRA, 36. Man/GalNAc-specific agglutinins/ TL C II, 37.Lectin (N-acetylglucosamine), 38. Lectin (N-acetylglucosamine), 39.Lectin (N-acetylglucosamine), 40. Lectin (N-acetylglucosamine), 41. Lectin (N-acetylglucosamine), 42. Ferruginol, 43.dehydroabieta-7-one cryptojaponol, 44. 8a-hydroxyabieta-9(11),13-dien-12-one, 45. 7a-hydroxydeoxycryptojaponol, 46. 6,7-dehydroroyleanone, 47.3a,12-diacetoxyabieta-6,8,11,13-tetraene, 48. pinusolidic acid, 49.forskolin, 50.α –cadinol, 51.betulinicacid, 52. betulonic acid, 53. Savinin, 54.honokiol, 55.magnolol, 56.supernatant of Cibotium barometz, 57.dried rhizome of Gentiana scabra, 58 tuber of Dioscorea batatas, 59. dried seed of Cassia tora, 60. dried stem, with leaf of Taxillus chinensis, 61. Forsythiae Fructus, 62. Scutellariae Radix, 63. Astragali Radix, 64. Bupleuri Radix, 65. Glycyrrhizae Radix, 66. Cinnamomi Cortex (CCE), 67. Ethanol extract of CC (Fr.1), 68. Butanol fraction of CC (Fr.2), 69. Aqueous fraction of CC (Fr.3), 70. Ethylacetate fraction of CC (Fr.4), 71. Caryophylli Flos (CFE), 72. Ethanol extract of CF (Fr.1), 73. Butanol fraction of CF (Fr.2), 74. Aqueous fraction of CF (Fr.3), 75. Ethylacetate fraction of CF (Fr.4), 76. Arteether, 77. artemether, 78. artemisicacid, 79. artemisinin, 80. artemisone, 81. dihydroartemisinin, 82. artesunate, 83. arteannuin, 84. lumefantrine, 85. andrographolide, 86. andrographolide, 87. andrographolide, 88. andrographolide, 89. andrographolide, 90. andrographolide, 91. Phillyrin (KD-1), 92. Liu Shen capsule, 93. Griffithsin, 94. Glycyrrhizin, 95. *L. nobilis*, 96. *T. orientalis*, 97. *J. oxycredrus* ssp, 98. Pyramidalis, 99. *P. palaestina*, 100. *P. palaestina*, 101. *S. officinalis*, 101. Glycyrrhizin

Of the plant compounds against the SARS-COV strain, Andrographolide had the highest SI. The same compound had the highest SI on SARS-COV-2 (Fig. 2). Then in order honokiol, 7a-hydroxydeoxycryptojaponol, Lycoris radiata, Extract/Amaryllidaceae and Lectin (Man-specific agglutinins) (APA) had the highest SI on SARS-COV strain.

Among the compounds acting on the PEDV strain, Oleanane triterpenes6 showed the highest SI = 44.54, followed by Oleanane triterpenes9. Among the compounds acting on IBV, the ethanolic extract of Lamiaceae showed the highest SI (Fig. 2).

The EC50 (Table 2), was reported in articles with two units of µg/ml and µM/L, and therefore we divide the articles into two groups according to the reported unit in our studies. In studies that investigated the EC50 of plant composition on SARS-COV and reported the result as µg/ml Lectin (Man-specific agglutinins) (EC50 = 0.45 ± 0.08 (µg/ml), Griffithsin (EC50 = 0.61 µg/ml), Mannose-specific agglutinins (EC50 = 1.6 ± 0.5 (µg/ml) and GlcNAc-specificictc Nictaba agglutinins (EC50 = 1.7 ± 0.3 (µg/ml), (GlcNAc) n-specific agglutinins UDA (EC50 = 1.3 ± 0.1 (µg/ml), extract of Amaryllidaceae (EC50 = 2.4 (± 0.2) (µg/ml) and extract of Lycoris radiate (EC50 = 2.1 (± 0.2) (µg/ml) have the lowest EC50.

Among the compounds that reported EC50 in µM/L units were 7â- hydroxydeoxycryptojaponol (EC50 = 1.15 µM/L), 8α-hydroxyabieta, 9 (11), 13-dien-12-one (EC50 = 1.47), 3α -12Diacetoxyabieta-6,8,11,13-tetraen (EC50 = 1.57) and Savinin (EC50 = 1.13) showed the lowest EC50. Silvestrol (EC50_HCOV_ = 0.003 µM/L, EC50_MERS-COV-2_ = 0.0013 µM/L) showed the lowest EC50 among the compounds that affected H-COV and MERS-COV-2. Among the compounds acting on the PEDV strain, Oleanane triterpenes8 (EC50 = 0.06 ± 0.02 (µM/L) showed the lowest EC50 (Table 2).

### Quality control

Quality control of 36 articles was reviewed using 20 items (Table 3). Study design features that help reduce bias, such as randomization, blindness of the test taker, reason for removing samples, how to select sample size, adjustments for multiple comparisons, similarity of variance between groups, cell authentication and cell contamination, cell strain confirmation, estimate of variation is reported within each group of data, and similarity of variance between the compared groups have not been reported in the literature. Only 52% of the articles reported the item "t or z test reported as one sided or two sided".

**Table 3:** Articles score based on Agency for Healthcare Research and Quality’s Methods Guide for Effectiveness of Reviews

Author/ Year	1	2	3	4	5	6	7	8	9	10	11	12	13	14	15	16	17	18	19	20
Christin Müller/ 2017	N	N	N	N	Y	Y	Y	Y	Y	N	N	N	Y	Y	N	N	N	Y	N	N
Pei-Win Cheng/ 2006	N	N	N	N	Y	Y	Y	Y	Y	Y	Y	N	Y	Y	N	N	NA	Y	N	N
Chung-Jen Chena/2008	N	N	N	N	Y	Y	Y	Y	Y	N	N	N	Y	Y	N	N	NA	Y	N	N
Shi-you Li/ 2005	N	N	N	N	Y	Y	Y	Y	Y	N	N	N	Y	Y	N	N	NA	Y	N	N
Jing-Ru Weng/2019	N	N	N	N	Y	Y	Y	Y	Y	Y	N	N	Y	Y	N	N	NA	Y	N	N
Els Keyaerts/ 2007	N	N	N	N	Y	Y	Y	Y	Y	N	N	N	Y	Y	N	N	NA	Y	N	N
Hye-Young Kim/ 2008	N	N	N	N	Y	Y	Y	Y	Y	N	N	N	Y	Y	N	N	N	Y	N	N
Hye-Young Kim/ 2010	N	N	N	N	Y	Y	Y	Y	Y	Y	N	N	Y	Y	N	N	NA	Y	N	N
Yohichi Kumaki/ 2011	N	N	N	N	Y	Y	Y	Y	Y	Y	Y	N	Y	Y	N	N	NA	Y	N	N
Raimundas Lelešius/ 2019	N	N	N	N	Y	Y	Y	Y	Y	Y	Y	N	Y	Y	N	N	NA	Y	N	N
K.H. Chiow/ 2015	N	N	N	N	Y	Y	Y	Y	Y	N	N	N	Y	Y	N	N	NA	Y	N	N
Jianguo Liang/2013	N	N	N	N	Y	Y	Y	Y	Y	N	N	N	Y	Y	N	N	NA	Y	N	N
Martin Michaelis/ 2011	N	N	N	N	N	Y	Y	Y	Y	N	N	N	Y	Y	N	N	NA	Y	N	N
Chih-Chun Wen/ 2007	N	N	N	N	Y	Y	Y	Y	Y	N	N	N	Y	Y	N	N	NA	Y	N	N
Chih-Chun Wen/ 2011	N	N	N	N	Y	Y	Y	Y	Y	N	N	N	Y	Y	N	Y	NA	Y	N	N
Jun-Li Yang/2015	N	N	N	N	Y	Y	Y	Y	Y	Y	Y	N	Y	Y	N	N	NA	Y	N	N
Jun-Li Yang/ 2015	N	N	N	N	Y	Y	Y	Y	Y	Y	Y	N	Y	Y	N	N	N	Y	N	N
Jiechao Yin/2011	N	N	N	N	Y	Y	Y	Y	Y	Y	Y	N	Y	Y	N	N	NA	Y	N	N
Christie Chen/ 2014	N	N	N	N	Y	Y	Y	Y	Y	N	N	N	Y	Y	N	N	NA	Y	N	N
Aarthi Sundararajan/2010	N	N	N	N	Y	Y	Y	Y	Y	Y	Y	N	Y	Y	N	N	NA	Y	N	N
Jean K. Millet/ 2016	N	N	N	N	Y	Y	Y	Y	Y	Y	Y	N	Y	Y	N	N	N	Y	N	N
Min Zhuang/ 2009	N	N	N	N	Y	Y	Y	Y	Y	N	N	N	Y	Y	N	N	N	Y	N	N
Nair/2020	N	N	N	N	Y	Y	Y	Y	Y	Y	N	N	Y	Y	N	N	NA	Y	N	N
Tsai/ 2020	N	N	N	N	Y	Y	Y	Y	Y	Y	Y	N	Y	Y	N	N	NA	Y	N	N
Ruiyuan Cao/ 2020	N	N	N	N	Y	Y	Y	Y	Y	Y	Y	N	Y	Y	N	N	N	Y	N	N
Sa-ngiamsuntorn/ / 2020	N	N	N	N	Y	Y	Y	Y	Y	N	N	N	Y	Y	N	N	N	Y	N	N
Bram M. ter Ellen/2021	N	N	N	N	Y	Y	Y	Y	Y	Y	Y	N	Y	Y	Y	N	NA	Y	N	N
Ka-Tim Choy/2020	N	N	N	N	Y	Y	Y	Y	Y	N	N	N	Y	Y	N	N	NA	Y	N	N
Qinhai Ma/2020	N	N	N	N	Y	Y	Y	Y	Y	Y	Y	N	Y	Y	N	N	N	Y	N	N
Qinhai Ma/2020	N	N	N	N	Y	Y	Y	Y	Y	Y	Y	N	Y	Y	N	N	NA	Y	N	N
Barry R. O’Keefe/ 2009	N	N	N	Y	Y	Y	Y	Y	Y	Y	Y	Y	Y	Y	N	N	NA	Y	N	N
Quanjie LI/2020	N	N	N	N	Y	Y	Y	Y	Y	N	N	N	Y	Y	N	N	NA	Y	N	N
Wael H. Roshdy/ 2020	N	N	N	N	Y	Y	Y	Y	Y	Y	Y	N	Y	Y	N	N	NA	Y	N	N
J Cinatl/2003	N	N	N	Y	Y	Y	Y	Y	Y	N	N	N	Y	Y	N	N	NA	Y	N	N
Dong Eon Kim/2019	N	N	N	N	Y	Y	Y	Y	Y	Y	Y	Y	Y	Y	N	N	NA	Y	N	N
Monica R. Loizzo/ 2008	N	N	N	N	Y	Y	Y	Y	Y	N	N	N	Y	Y	N	N	NA	Y	N	N
Percentage	0	0	0	0	97	100	100	100	100	90	52	5	100	100	2	0	0	100	0	0

1) samples were excluded from the analysis, 2) which method of randomization was used to determine how samples were allocated to experimental groups, 3) whether the investigator was blinded, 4) how the sample size was chosen 5)The exact sample size (6) whether the samples represent technical or biological replicates, 7) how many times the experiment shown was replicated, 8)The summary estimates are defined as a median or average, 9) The error bars are defined as sd., sem. or ci., 10) Common statistical test, or the test is described, 11) t or z test reported as one sided or two sided, 12) Adjustments for multiple comparisons are applied, 13) The statistical test results are presented, 14)The authors show that their data meet the assumptions of the tests, 15)An estimate of variation is reported within each group of data, 16) The variance is similar between the groups that are being statistically compared, 17) antibody citation, catalog number, 18) The source of cell lines, 19) whether the cell lines used have been authenticated recently, 20) whether the lines used have been tested for contamination recently

All articles have reported the following: the exact sample size, whether the samples represent technical or biological replicates, how many times the experiment shown was replicated, the summary estimates are defined as a median or average, the error bars are defined as s.d., s.e.m. or c.i., Common statistical test, or the test is described, the statistical test results are presented, the authors show that their data meet the assumptions of the tests and the source of cell lines.

### Virus inhibition

**Fig. 3:**
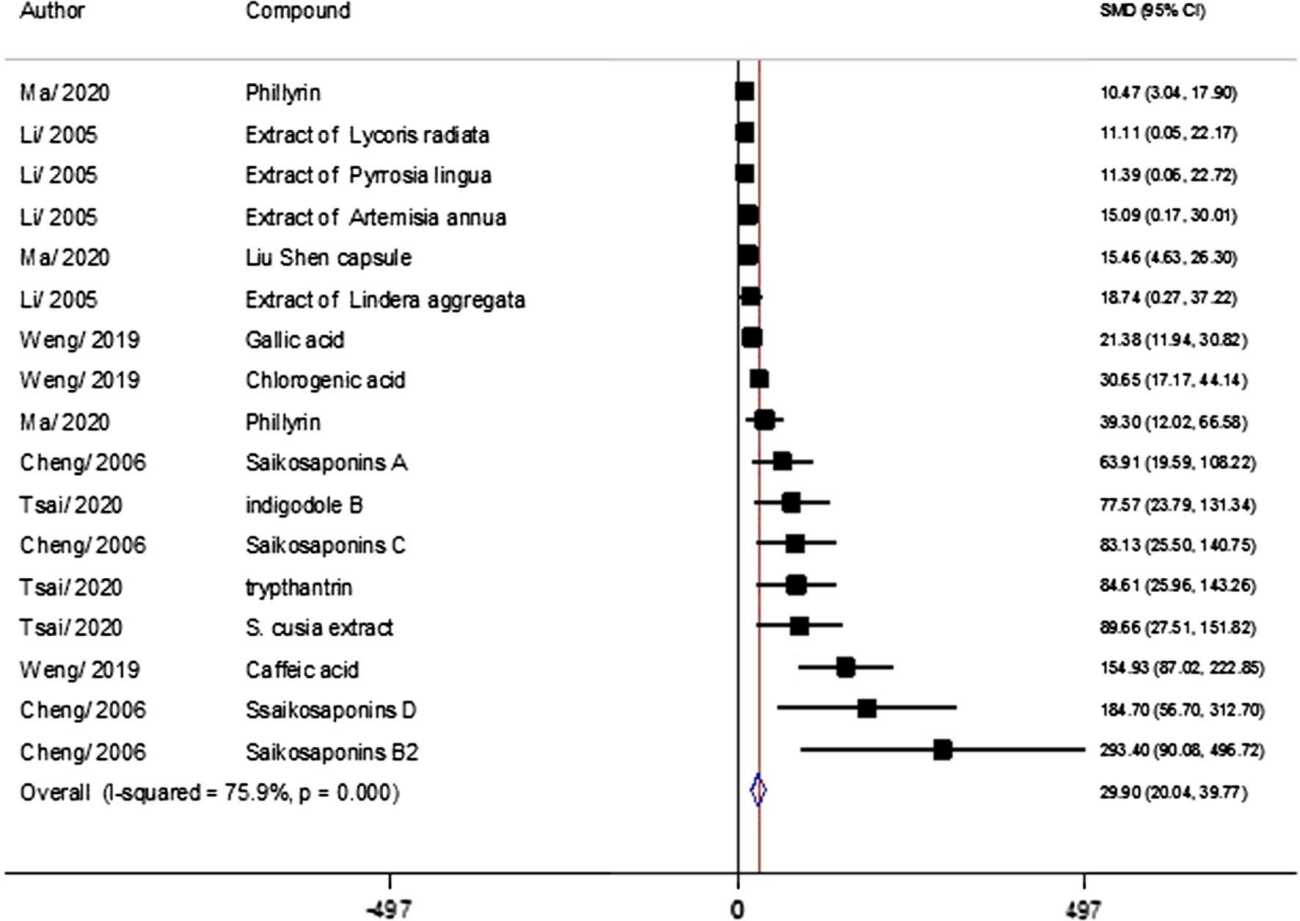
Forest plot of virus inhibition from studies

The effect of herbal compound on the virus inhibition showed (Fig. 3) that Saikosaponins B2 (SMD = 293.4; 95% CI 90.08–496.72), Saikosaponins D, Caffeic acid, and *S. cusia* extract inhibit virus growth more than other compounds. Subgroup studies was performed to find the source of heterogeneity among studies (I^2^ = 75.9, *p* < 0.0001).

All three factors, including chemical structure, virus strain, and host cell type, are heterogeneous agents. We subgrouped the data based on chemical structure into groups of phenolic compounds (9 experiment), alkaloids (2 experiment) and plant extracts (6 experiment) (Table 4). Antiviral effect on alkaloid compounds 80.78% (ES = 80.78; 95% CI 41.14 to 120.41; < 0.0001), phenolic compounds (ES = 44.85; 95% CI 26.17 to 63.53; < 0.0001), and extracts (ES = 14.59; 95% CI 7.96–21.22; < 0.0001) decreases, respectively.

**Table 4:** Results of subgroup analysis based on various variables for virus titer outcome

Subgroup	Number of experiments	Heterogeneity (p value)	ES (95% CI)	*p* value
Chemical structure				
Phenolic compound	7	84.7% (< 0.0001)	− 7.40 (− 10.81 to − 3.97)	< 0.0001
Lectin	3	29.4% (< 0.24)	− 18.36 (− 26.60 to − 10.88)	< 0.0001
Extract of plant	4	84.3% (< 0.0001)	− 21.83 (− 37.83 to − 5.84)	0.007
Alkaloid	2	53.5% (0.143)	− 27.18 (− 48.84 to − 5.39)	0.014
Peptide	4	23.1%(0.27)	− 10.235(− 14.73 to − 5.74)	< 0.0001
Virus strain				
MERS-COV-2	3	70.8% (< 0.0001)	− 10.50 (− 18.91 to − 2.10)	0.014
HcoV	8	79.1% (< 0.0001)	− 17.00 (− 23.36 to − 10.64)	< 0.0001
SARS-COV-2	9	80.3% (< 0.0001)	− 9.70 (− 14.23 to − 5.175)	< 0.0001
Cell line				
Human	10	79.5% (< 0.0001)	− 8.96 (− 12.56 to − 5.35)	< 0.0001
Monkey	10	75.7% (< 0.0001)	− 15.22 (− 20.31 to − 10.13)	< 0.0001

If the data were grouped by virus strain, the effect of plant compounds on HCoV (ES = 71.92; 95% CI 46.63–97.21; < 0.0001) was greater than that of SARS-COV-2 strains (ES = 15.81; 95% CI5.44) to 26.19; *p* = 0.003) and SARS-COV (ES = 12.92; 95% CI 6.38–19.46; < 0.0001). In data grouping by cell type, the effect of plant compounds on cells of human origin (ES = 109.98; 95% CI 45.53–174.43; < 0.001) was greater than that of cells of monkey origin (ES = 23.70; 95% CI 15.07–32.33; < 0.0001).

### Virus titer

**Fig. 4:**
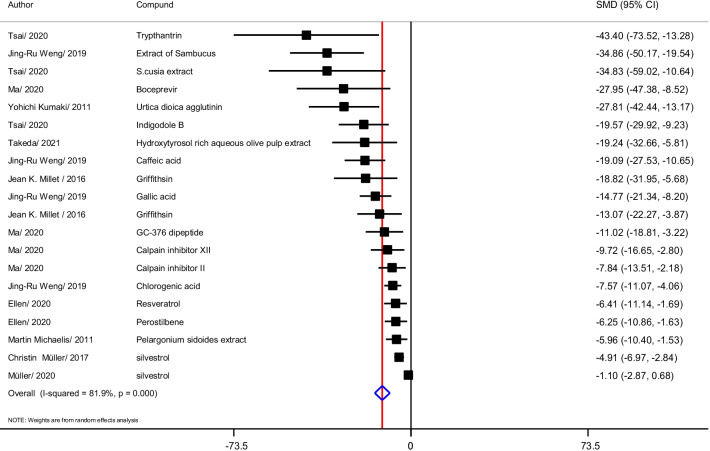
Forest chart for studies that measured virus titers

Virus titer analysis after treatment with herbal medicine in 10 articles and 20 studies showed (Fig. 4) that Trypthantrin (SMD = − 43.40; 95% CI − 73.52 to − 13.28), Sambucus extract, *S. cusia* extract, Boceprevir, Urtica dioica agglutinin, Indigole B, Hydroxytyrosol aqueus olive pulp, Caffeic acid, Griffithsin, Gallic acid had the most effects on reducing the virus titer, respectively. The effect of the other compounds is shown in the Fig. 4. Heterogeneity of studies was 81.9% I^2^ = 81.9%, *p* < 0.0001).

The data was grouped based on the chemical structure into groups of phenolic compounds, alkaloids, peptides and lectins. The effect of alkaloid compounds (ES = − 27.18; 95% CI − 48.84 to − 5.39; 0.014), extract Plant (ES = − 21.83; 95% CI − 37.83 to − 5.84; 0.007), Lectin compounds (ES = − 18.36; 95% CI − 26.60 to − 10.88; < 0.0001), Peptide compounds (ES = − 10.235; 95% CI − 14.73 to − 5.74; < 0.0001) and phenolic compounds (ES = − 7.40; 95% CI − 10.81 to − 3.97; < 0.0001) decrease on virus titer, respectively.

The data was grouped by virus strain, the effect of plant compounds on HCoV strains (ES = − 17.00; 95% CI − 23.36 to − 10.64; *p* < 0.0001) is greater than that of other strains on the SARS-COV strain. − 2 (ES = − 9.70; 95% CI − 14.23 to − 5.175; *p* < 0.0001) and the MERS-COV-2 strain (ES = − 10.50; 95% CI − 18.91 to − 2.10; *p* = 0.014) are approximately equal. If the data grouped according to the type of host cell, the effect of compounds on the cells of monkey origin (ES = − 15.22; 95% CI − 20.31 to − 10.13; < 0.0001 have a greater effect compared to the cells of human origin (ES = − 8.96; 95% CI − 12.56 to − 5.35; < 0.0001).

## Discussion

**Fig. 5:**
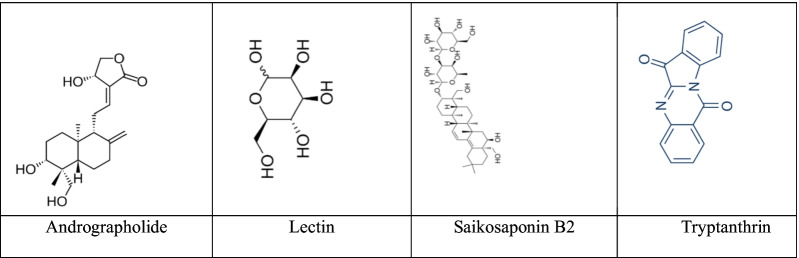
Structure of Andrographolide, Lectin, Saikosaponin B2, Tryptanthrin

According to the SI index, Silvestrol had the greatest effect on the coronavirus family. Among the compounds whose effects on SARS-COV-2 were investigated, Andrographolide (Fig. 5A) had the highest effect. Andrographolide is a diterpene lactone in the isoprenoid family, which is recognized for its broad-spectrum antiviral activity [46]. I*n silico* studies predicted Andrographolide has a potent anti-SARS-COV-2 activity through specific aiming of the host ACE2 receptor and viral factors, such as RNA-dependent RNA polymerase, main protease, 3-CL protease, PL protease, and spike protein [16, 21, 44]. Recently, Shi et al. demonstrated an inhibitory effect of Andrographolide against SARS-COV-2 main protease (Mpro) [47].

Based on the EC50 index, Lectin (Fig. 5B), Griffithsin and 7a-hydroxydeoxycryptojaponol showed the lowest levels. Plant lectins have significant antiviral properties against coronaviruses and are non-toxic for host cells. The strongest anti-coronavirus activity was found predominantly among the mannose-binding lectins. The first target in the replication cycle of SARS-COV is located in probably viral attachment, and the second target is at the end of the infectious virus cycle [20]. Lectins are the sparkle of hope for fighting coronaviruses and the worldwide COVID 19 [1].

The results of meta-analysis of inhibiting the growth of the virus after treatment with herbal medicine showed that among the herbal compounds, the antiviral effect of the alkaloid compound Saikosaponin B2 (Fig. 5C) is the most. Saikosaponin B2 showed strong potent anti-coronaviral activity and its method of action probably involves interference in the early stage of viral replication, such as virus uptake and penetration [9]. The results of the virus titer also confirmed Tryptanthrin alkaloid compound (Fig. 5D) as the strongest antiviral effect. Tryptanthrin prevented the both early and the late stages of coronaviral replication, principally by blocking viral RNA genome synthesis and Papain-like protease2 activity [48].

Studies by other researchers have shown that alkaloids, as one of the most widely used natural compounds, can be an effective treatment against SARS-COV-2 due to their simultaneous effects on several therapeutic targets with prominent antiviral effects [34].

## Conclusion

Due to the multiplicity of study methods, definitive conclusions are not possible. However, in this study, we tried to gather all available evidence on the effect of plant compounds on SARS-COV-2 to be used for the development and use of promising antiviral agents against SARS-COV-2 and other coronaviruses.

According to the SI results, Silvesterol had the greatest effect on the coronavirus family and Andrographolide had the greatest effect on SARS-COV-2. Based on the EC50, Lectin, Griffithsin and 7a-hydroxydeoxycryptojaponol showed the lowest levels. The results of meta-analysis confirmed the growth inhibition of Saikosaponin B2 and the virus titer results confirmed the alkaloid compound Tryptanthrin as the strongest antiviral molecule. The small number of studies that used alkaloid was one of the limitations of this study and it is suggested to investigate the effect of more alkaloid compounds on coronavirus.

## Data Availability

Data are available from corresponding author (FR) by reasonable request.
